# Exploring the Utility of Google Mobility Data During the COVID-19 Pandemic in India: Digital Epidemiological Analysis

**DOI:** 10.2196/29957

**Published:** 2021-08-30

**Authors:** Kamal Kishore, Vidushi Jaswal, Madhur Verma, Vipin Koushal

**Affiliations:** 1 Postgraduate Institute of Medical Education and Research Chandigarh India; 2 Mehr Chand Mahajan DAV College Chandigarh India; 3 All India Institute of Medical Sciences Bathinda India

**Keywords:** COVID-19, lockdown, nonpharmaceutical Interventions, social distancing, digital surveillance, Google Community Mobility Reports, community mobility

## Abstract

**Background:**

Association between human mobility and disease transmission has been established for COVID-19, but quantifying the levels of mobility over large geographical areas is difficult. Google has released Community Mobility Reports (CMRs) containing data about the movement of people, collated from mobile devices.

**Objective:**

The aim of this study is to explore the use of CMRs to assess the role of mobility in spreading COVID-19 infection in India.

**Methods:**

In this ecological study, we analyzed CMRs to determine human mobility between March and October 2020. The data were compared for the phases before the lockdown (between March 14 and 25, 2020), during lockdown (March 25-June 7, 2020), and after the lockdown (June 8-October 15, 2020) with the reference periods (ie, January 3-February 6, 2020). Another data set depicting the burden of COVID-19 as per various disease severity indicators was derived from a crowdsourced API. The relationship between the two data sets was investigated using the Kendall tau correlation to depict the correlation between mobility and disease severity.

**Results:**

At the national level, mobility decreased from –38% to –77% for all areas but residential (which showed an increase of 24.6%) during the lockdown compared to the reference period. At the beginning of the unlock phase, the state of Sikkim (minimum cases: 7) with a –60% reduction in mobility depicted more mobility compared to –82% in Maharashtra (maximum cases: 1.59 million). Residential mobility was negatively correlated (–0.05 to –0.91) with all other measures of mobility. The magnitude of the correlations for intramobility indicators was comparatively low for the lockdown phase (correlation ≥0.5 for 12 indicators) compared to the other phases (correlation ≥0.5 for 45 and 18 indicators in the prelockdown and unlock phases, respectively). A high correlation coefficient between epidemiological and mobility indicators was observed for the lockdown and unlock phases compared to the prelockdown phase.

**Conclusions:**

Mobile-based open-source mobility data can be used to assess the effectiveness of social distancing in mitigating disease spread. CMR data depicted an association between mobility and disease severity, and we suggest using this technique to supplement future COVID-19 surveillance.

## Introduction

Infectious diseases have caused profound disruptions throughout the history of humanity. Despite a decrease in the number of deaths attributed to contagious diseases, there has been a constant rise in the number of outbreaks over the past few years due to emerging and re-emerging infectious agents [1]. Influenza, dengue fever, and HIV/AIDS are the three leading contagious diseases that have infected millions of people globally [2]. In addition to these, approximately 215 different infectious agents have caused 12,102 outbreaks in 219 countries over the last 30 years [3]. In general, there have been significant advances in the treatment and curing of infectious diseases. However, infectious diseases pose a considerable challenge to the health system due to their frequency, infectivity, and mobility in today’s extensively interconnected world. Therefore, early detection and prevention of infectious diseases continues to be a top priority among the global health community.

The current COVID-19 pandemic has disrupted and overwhelmed health systems worldwide. COVID-19 is an infectious disease that is caused by a newly discovered coronavirus, ﻿and the main route of transmission is thought to be through respiratory droplets [4]. The index case of COVID-19 was traced to December 1, 2019, in Wuhan, China [5]. The aggressive nature of the spread of COVID-19 led to its declaration as a “public health emergency of international concern” and then a pandemic by the World Health Organization (WHO) on January 30 and March 11, 2020, respectively. As per the WHO, 216 countries had reported more than 121 million cases and 2.6 million deaths due to COVID-19 as of March 17, 2021 [6]. There were 11.4 million confirmed cases in India alone, with 0.16 million deaths, and it is among the most severely affected countries to date [7]. Due to a lack of effective treatment strategy, nonpharmaceutical interventions (NPIs), such as restricted mobility, home quarantine, and lockdown measures, were enforced worldwide to halt interhuman transmission of the virus [8]. As India is the second most populous country in the world, with suboptimal investment, NPIs were seen as the most crucial part of pandemic mitigation. Hence, the Government of India also implemented a countrywide lockdown to halt disease progression on March 24, 2020 [9].

Research has demonstrated the association between mobility and disease transmission for various infectious diseases, such as cholera, dengue, influenza, Ebola, malaria, measles, and COVID-19 [10-17]. NPIs are intended to slow the rapid disease transmission and contain the disease burden until effective pharmacological management options become accessible [18,19]. Implementing NPIs in response to infectious disease outbreaks is not a new method to limit mobility; they have been used for centuries [20,21]. More recently, such measures were implemented during the containment of the severe acute respiratory syndrome (SARS) and Middle East respiratory syndrome (MERS) epidemics, which occurred in the last decades [22,23].

Although the connection between mobility and disease has been known for centuries, establishing this causal association is challenging, as measuring and quantifying the levels of mobility at the population level is difficult. This can be attributed to the challenges in obtaining access to mobility and disease data. However, numerous mathematical models have demonstrated such associations between mobility and infectious disease transmission dynamics [24-26]. Moreover, during the current pandemic, the digital ecosystem has supplemented traditional surveillance to provide data about disease severity and mobility in real time.

Given the highly infectious nature of COVID-19, the importance of digital epidemiology could be felt in disease containment [24-27]. Digital epidemiology is a branch of epidemiology that uses data generated outside the public health system [28]. Google Flu and Google Trends have been successfully used to study various communicable and noncommunicable diseases [29,30]. On similar lines, Google released Community Mobility Report (CMR) data collated from people who accessed its applications using mobile and handheld devices. The restriction in mobility by the Indian government and the data availability provides researchers with an opportunity to empirically study the relationship between social activity, mobility, and COVID-19 incidence. However, there is a shortage of scientific literature that documents the use of these data for surveillance purposes. Very few researchers have tried to correlate mobility trends with the aggressiveness of the disease [31-34]. Sulyok and Walker [31] depicted negative correlations between CMR data and case incidence for major industrialized countries of Western Europe and North America. Wang and Yamamoto [32] also depicted that a ﻿model using CMR data can describe the combined effects of mobility at the local level and human activities on the transmission of COVID-19. Cot et al [33] analyzed Google and Apple mobility data. They concluded that a substantial decrease in the infection rate occurred 2-5 weeks after the onset of mobility reduction [33]. None of these studies explored the association of mobility with any other epidemiological indicators except disease incidence; meanwhile, it has been established that disease incidence alone is not an ideal measure for making comparisons [35]. Therefore, in this study, we attempt to understand and explore the role of mobility in spreading COVID-19 infection in India using mobility data from Google. During the pandemic, the central government has issued various health advisories; however, because health is a state responsibility, the final implementation of those instructions depends on the state itself. Therefore, we hypothesized that the states with strict enforcement of lockdown would witness fewer cases and vice versa. Hence, we have also examined the states with the maximum and minimum numbers of cases for changes in mobility as per CMR data.

## Methods

### Study Design

In this ecological study, we analyzed secondary data available in the public domain between March 14 and October 16, 2020.

### Study Period

Many interventions were implemented in India at the national and subnational levels during the lockdown period and were subsequently eased out in a phased manner. To begin, India issued travel advisories and restricted international travel between January and March 2020. By early March, when case numbers started to increase, states scaled up movement restrictions. On March 25, India entered a nationwide lockdown to ramp up preparedness [36]. The mobility data were assessed for three significant periods, based on the implementation of social mobility restrictions by the Indian government to mitigate the pandemic [37]. Robust data for COVID-19 disease burden were available in the public domain from March 14, 2020. Hence, the three phases were labeled as prelockdown (March 14-24, 2020), lockdown (March 25-June 7, 2020), and unlock (June 8-October 15, 2020).

### Data Sources

#### COVID-19 Data

The data sets for COVID-19 cases in India were crowdsourced and made freely available through an API by a volunteer group. The API maintains the records of confirmed, active, recovered, and deceased people for all the Indian states and union territories. The data in the API are gathered daily using state bulletins and official handles. After the data are validated, they are made available daily through Google Sheets [7].

#### Mobility Data

Google collects and stores individuals’ commuting information through a GPS linked to Google Maps. These data are made available on the web in the public domain, after aggregating and anonymizing personally identifiable information, as “COVID-19 Community Mobility Reports” (Multimedia Appendix 1) [38]. A CMR compares the changes in activity and mobility during and after lockdown compared to before lockdown. At the start of the study, the mobility data for 135 countries were available from Google. The mobility data for India have been made available at the state and union territory levels since February 15, 2020. Multimedia Appendix 1 contains further details about this website. The CMR provides the percentage changes in activity for 6 key categories (groceries and pharmacies, parks, transit, retail and recreation, residential, and workplaces) compared to the baseline days before the advent of COVID-19 (5 weeks, from January 3 to February 6, 2020) [39]. Daily activity changes are compared to the corresponding baseline figure day. For example, data on Monday are compared to corresponding data from the baseline series for a Monday. Baseline day figures are calculated for each day of the week for each country and are calculated as the median value [38]. The values represent the relative changes in percentage compared to the baseline days, not the absolute number of visitors. For instance, a value of –50 in the workplaces data set on a Monday indicates a 50% drop compared to the Monday in the reference period. Similarly, a positive value indicates an increase in mobility compared to the reference period.

### Primary Outcome Variables and Covariates

The frequency of daily infected cases, deaths, and recovered cases were the primary variables of this study. The disease burden data for India by individual states and union territories were depicted in cases per million (CPM), case fatality rate (CFR), and doubling rate (DR), which were calculated using the standard formulae [40-42]. We used census population data from the different states of India as a reference [43]. The mobility indicators pointing toward disease spread were the covariates of interest. A CMR provides data for 6 mobility indicators, used as covariates, which give information on people’s movement. It was significant to assess the variability in people’s mobility during the unlocking phase in response to the caseload of each state during the lockdown. For the principle of parsimony, we report the frequency of cases using the median and range values for the states with the maximum and minimum numbers of cases.

### Data Analysis

We downloaded the mobility and COVID-19 data in the .csv format on October 16, 2020, and we replaced the state codes for the India COVID-19 data with state names using metadata. The mobility data at the national and state levels were filtered and stored. Subsequently, we merged the mobility and COVID-19 data for India and the respective states and union territories using the date variable and created a new spreadsheet. Finally, we arranged the data in separate spreadsheets for the national and state levels for further analysis. Subsequently, the relationship between mobility and COVID-19 spread for the prelockdown and unlock phases was investigated using the Kendall tau correlation. This approach is more general and consistent with the ranking system and is proportional to the number of concordant pairs minus the number of discordant pairs. The value of tau ranges from +1 to –1 for identically and oppositely ranking pairs, respectively. Because it is an initial empirical investigation of the relationship between mobility and epidemiological indicators, the emphasis is on the magnitude of the correlation rather than the *P* value. Further, we calculated and reported the 95% CI with all the point estimates to provide readers with an idea of the estimate range.

### Ethical Clearance

Ethical clearance for the study was obtained from the Institutional Review Board of the Postgraduate Institute of Medical Education and Research, Chandigarh, India, vide letter INT/IEC/2020/SPL-1594.

## Results

### Disease Burden During Different Phases of Lockdown

**Figure 1:**
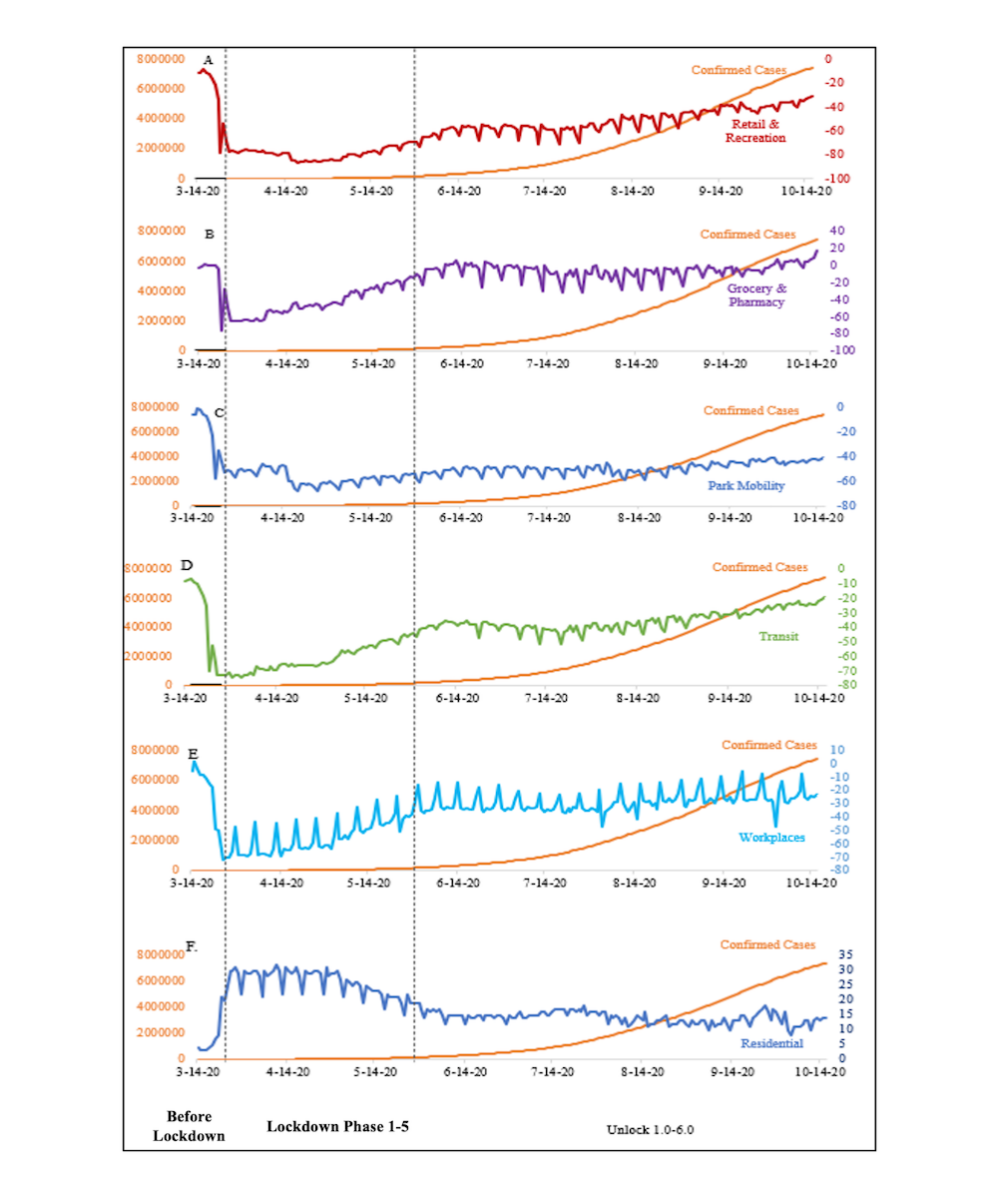
Line diagram depicting COVID-19 cases and mobility trends for India from March 14 to October 14, 2020.

The line graphs display the mobility trend and rise in the number of cases during these phases (Figure 1). At the end of phase 1, as in, just before the national lockdown, the numbers of cumulative cases, cumulative deaths, and cumulative recoveries throughout India were recorded to be 567, 40, and 10, respectively. The lockdown was enforced for 75 days, until June 8, 2020, but the surge in the cumulative caseloads continued (Table 1). This was followed by sequential unlocking, after which a further surge was witnessed. As of October 15, 2020 (second unlock phase), the reported numbers of cumulative cases, cumulative deaths, and cumulative recoveries in India surpassed 7.5 million, 0.1 million, and 6.6 million, respectively, with marked interstate variations.

**Table 1:** Statewise burden (cumulative total) of the COVID-19 pandemic in India during the different phases of the lockdown (March-October 2020).

Region and state in India			Number of cases in each phase of lockdown, n (%)																					
			Prelockdown (before March 25, 2020)							Lockdown (March 25-June 7, 2020)							Unlock (June 8-October 15, 2020)							
		C^a^		D^b^		R^c^		C			D		R		C			D		R				
India			567 (100)		10 (100)		40 (100)		257,478 (100)			7205 (100)		123,848 (100)		7,546,965 (100)			114,042 (100)		6,658,418 (100)			
**North**																								
	Delhi	30 (5.3)		1 (10)		6 (15)		28,936 (11.2)			812 (11.3)		10,999 (8.9)		331,017 (4.4)			6009 (5.3)		301,716(4.5)				
	Haryana	30 (5.3)		0 (0)		11 (27.5)		4448 (1.7)			28 (0.4)		1473 (1.2)		150,033 (2.0)			1640 (1.4)		138,350(2.1)				
	Punjab	29 (5.1)		1 (10)		0 (0)		2608 (1)			51 (0.7)		2106 (1.7)		127,154 (1.7)			3999 (3.5)		116,925(1.8)				
	Jammu and Kashmir	6 (1.1)		0 (0)		0 (0)		4087 (1.6)			41 (0.6)		1216 (1)		87,942 (1.2)			1379 (1.2)		77,886 (1.2)				
	Uttarakhand	4 (0.7)		0 (0)		0 (0)		1355 (0.5)			13 (0.2)		528 (0.4)		58,024 (0.8)			927 (0.8)		50,982 (0.8)				
	Himachal Pradesh	3 (0.5)		1 (10)		0 (0)		411 (0.2)			6 (0.1)		219 (0.2)		18,967 (0.3)			263 (0.2)		16,038 (0.2)				
	Chandigarh	7 (1.2)		0 (0)		0 (0)		314 (0.1)			5 (0.1)		274 (0.2)		13,646 (0.2)			208 (0.2)		12,554 (0.2)				
	Ladakh	13 (2.3)		0 (0)		0 (0)		103 (0)			1 (0)		50 (0)		5598 (0.1)			66 (0.1)		4615 (0.1)				
**Central**																								
	Uttar Pradesh	35 (6.2)		0 (0)		11 (27.5)		10,536 (4.1)			275 (3.8)		6185 (5)		455,146 (6.0)			6658 (5.8)		415,592(6.2)				
	Rajasthan	32 (5.6)		0 (0)		3 (7.5)		10,599 (4.1)			240 (3.3)		7754 (6.3)		173,266 (2.3)			1747 (1.5)		150,379(2.3)				
	Chhattisgarh	1 (0.2)		0 (0)		0 (0)		1073 (0.4)			4 (0.1)		266 (0.2)		160,396 (2.1)			1478 (1.3)		132,168(2)				
	Madhya Pradesh	7 (1.2)		0 (0)		0 (0)		9401 (3.7)			413 (5.7)		6331 (5.1)		160,188 (2.1)			2774 (2.4)		144,134(2.2)				
**West**																								
	Maharashtra	107 (18.9)		2 (20)		0 (0)		85,975 (33.4)			3059 (42.5)		39,314 (31.7)		1,595,381 (21.1)			42,114 (36.9)		1,369,810 (20.6)				
	Gujarat	34 (6)		1 (10)		0 (0)		20,097 (7.8)			1249 (17.3)		13,643 (11)		159,725 (2.1)			3637 (3.2)		141,753(2.1)				
	Goa	0 (0)		0 (0)		0 (0)		300 (0.1)			0 (0)		65 (0.1)		40,587 (0.5)			544 (0.5)		36,395 (0.5)				
	Dadra and Nagar Haveli	0 (0)		0 (0)		0 (0)		20 (0)			0 (0)		2 (0)		3176 (0)			2 (0)		3079 (0)				
	Daman and Diu	0 (0)		0 (0)		0 (0)		0 (0)			0 (0)		0 (0)		0 (0)			0 (0)		0 (0)				
**South**																								
	Andhra Pradesh	8 (1.4)		0 (0)		0 (0)		4659 (1.8)			75 (1)		2669 (2.2)		782,123 (10.4)			6425 (5.6)		739,307(11.1)				
	Karnataka	41 (7.2)		1(10)		3 (7.5)		5452 (2.1)			61 (0.8)		2132 (1.7)		765,586 (10.1)			9889 (8.7)		645,826(9.7)				
	Tamil Nadu	18 (3.2)		1(10)		1 (2.5)		31,667 (12.3)			272 (3.8)		16,999 (13.7)		687,400 (9.1)			10,642 (9.3)		637,637(9.6)				
	Kerala	109 (19.2)		0 (0)		4 (10)		1915 (0.7)			16 (0.2)		803 (0.6)		341,860 (4.5)			1162 (1)		245,394(3.7)				
	Telangana	37 (6.5)		0 (0)		1 (2.5)		3650 (1.4)			137 (1.9)		1742 (1.4)		221,601 (2.9)			1271 (1.1)		198,790(3)				
	Puducherry	1 (0.2)		0 (0)		0 (0)		119 (0)			0 (0)		49 (0)		33,143 (0.4)			574 (0.5)		28,290 (0.4)				
	Andaman and Nicobar Islands	0 (0)		0 (0)		0 (0)		33 (0)			0 (0)		33 (0)		4104 (0.1)			56 (0)		3859 (0.1)				
	Lakshadweep	0 (0)		0 (0)		0 (0)		0 (0)			0 (0)		0 (0)		0 (0)			0 (0)		0 (0)				
**East**																								
	West Bengal	9 (1.6)		1(10)		0 (0)		8187 (3.2)			396 (5.5)		3303 (2.7)		0 (0)			6056 (5.3)		281,053 (4.2)				
	Odisha	2 (0.4)		0 (0)		0 (0)		2856 (1.1)			11 (0.2)		1894 (1.5)		268,364 (3.6)			1188 (1.0)		246,837 (3.7)				
	Bihar	3 (0.5)		1(10)		0 (0)		5070 (2.0)			30 (0.4)		2405 (1.9)		204,212 (2.7)			996 (0.9)		192,594 (2.9)				
	Jharkhand	0 (0)		0 (0)		0 (0)		1103 (0.4)			7 (0.1)		490 (0.4)		96,327 (1.3)			39 (0.7)		89,011 (1.3)				
**Northeast**																								
	Assam	0 (0)		0 (0)		0 (0)		2682 (1.0)			3 (0)		637 (0.5)		200,607 (2.7)			872 (0.8)		171,680(2.6)				
	Tripura	0 (0)		0 (0)		0 (0)		802 (0.3)			0 (0)		192 (0.2)		29,465 (0.4)			326 (0.3)		26,199 (0.4)				
	Manipur	1 (0.2)		0 (0)		0 (0)		172 (0.1)			0 (0)		52 (0)		15,463 (0.2)			116 (0.1)		11,741 (0.2)				
	Arunachal Pradesh	0 (0)		0 (0)		0 (0)		48 (0)			0 (0)		1 (0)		13,348 (0.2)			30 (0)		10,315 (0.2)				
	Meghalaya	0 (0)		0 (0)		0 (0)		36 (0)			1 (0)		13 (0)		8404 (0.1)			75 (0.1)		6034 (0.1)				
	Nagaland	0 (0)		0 (0)		0 (0)		116 (0)			0 (0)		8 (0)		7816 (0.1)			21 (0)		6142 (0.1)				
	Sikkim	0 (0)		0 (0)		0 (0)		7 (0)			0 (0)		0 (0)		3610 (0)			60 (0.1)		3185 (0)				
	Mizoram	0 (0)		0 (0)		0 (0)		34 (0)			0 (0)		1 (0)		2253 (0)			0 (0)		2148 (0)				

^a^C: confirmed cases of COVID-19.

^b^D: deceased due to COVID-19.

^c^R: recovered from COVID-19.

### Disease Severity/Epidemiologic Indicators

Table 2 presents crucial epidemiologic indicators that were used to estimate disease burden in terms of the CPM, DR, and CFR of COVID-19. CPM increased to 40 (36.6-43.3) at the national level by the end of phase 3 (unlock). The disease DR also increased to 33.4 (30.3-36.5), while CFR—a vital indicator of the severity of the disease in an epidemic—decreased to 2.3% (95% CI 1.9%-2.6%) on October 15, 2020. The states of Punjab (4.6%, 95% CI 4.0%-5.3%) and Maharashtra (4.1%, 95% CI 3.4%-4.8%) reported the highest CFRs; meanwhile, Mizoram, Lakshadweep and Daman, and Diu reported CFRs of zero.

**Table 2:** Interstate comparison of COVID-19–related statistics during the different phases of lockdown in India (March-October 2020).

Region and state in India		Statistics in each phase of lockdown, mean (95% CI)										
		Prelockdown (before March 25, 2020)				Lockdown (March 25-June 7, 2020)				Unlock (June 8-October 15, 2020)		
		CPM^a^	DR^b^	CFR^c^	CPM		DR	CFR	CPM		DR	CFR
India		0 (0-0.1)	4.1 (2.8-5.5)	22.0 (0-47.6)	2.5 (2-3)		10.6 (9.6-11.5)	12 (9.7-14.3)	40 (36.6-43.3)		33.4 (30.3-36.5)	2.3 (1.9-2.6)
**North**												
	Delhi	0.1 (0-0.2)	2.3 (0-4.8)	0	20.6 (15.6-25.6)		12.6 (10.6-14.6)	12.6 (7.2-18)	121.4 (111.7-131)		58.5 (53-64)	2.8 (1.5-4.1)
	Haryana	0.1 (0-0.2)	4.3 (0-8.9)	0	2.1 (1.3-2.9)		20.9 (14.7-27.2)	4.3 (0.9-7.6)	38.8 (34.9-42.8)		35.4 (31.7-39.1)	1.5 (1.2-1.7)
	Punjab	0.1 (0-0.2)	2.3 (0-5.3)	0	1.1 (0.7-1.5)		50.8 (8-93.7)	12 (6.3-17.7)	31.2 (26.9-35.5)		35.9 (29.6-42.3)	4.6 (4-5.3)
	Jammu and Kashmir	0 (0-0.1)	0.8 (0-2.3)	0	4 (2.6-5.4)		14.4 (12.1-16.8)	5.9 (1.7-10.1)	46.3 (41.1-51.6)		37.8 (33.9-41.6)	2.6 (2.2-3)
	Uttarakhand	0 (0-0.1)	0.5 (0-1.3)	0	1.6 (0.8-2.4)		11.2 (7.6-14.7)	4.8 (0.4-10)	37.9 (31.9-43.8)		36.8 (31.3-42.3)	2 (1.7-2.4)
	Himachal Pradesh	0 (0-0.1)	0	10.0 (0-32.6)	0.7 (0.5-1)		11.5 (8-14.9)	2.9 (1.1-6.8)	18.7 (16-21.4)		32.4 (28.6-36.1)	1.5 (1.1-2)
	Chandigarh	0.5 (0.2-1.2)	0.5 (0-1.6)	0	3.5 (2.3-4.8)		24.7 (12.2-37.2)	8.8 (1.2-16.4)	86.5 (71.4-101.7)		39.9 (33-46.7)	3.3 (1.5-5)
	Ladakh	4.1 (1.7-9.9)	0	0	4.2 (1.5-6.8)		3.9 (1.4-6.4)	1.6 (1.6-4.8)	143 (123.6-162.3)		57.3 (43.8-70.8)	0
**Central**												
	Uttar Pradesh	0.1 (0.0-0.2)	6.2 (3.5-8.8)	0	0.6 (0.5-0.7)		13.7 (12-15.5)	6.7 (3-10.3)	14.1 (12.6-15.5)		35.5 (30.6-40.5)	2.3 (2-2.6)
	Rajasthan	0 (0-0.1)	2.5 (0.7-4.2)	0	1.7 (1.5-2)		14.6 (12.7-16.5)	7.3 (3-11.7)	15.1 (13.8-16.4)		35.3 (33.7-36.9)	1.5 (1.3-1.6)
	Chhattisgarh	0	0	0	0.5 (0.3-0.7)		7.4 (4-10.8)	0.4 (0.1-0.8)	40.7 (33.7-47.7)		22.4 (20.6-24.3)	1.5 (1.2-1.9)
	Madhya Pradesh	0.1	0.9 (0-2.5)	0	1.5 (1.2-1.7)		18.2 (12.7-23.7)	16.1 (10.4-21.8)	13.3 (11.6-14.9)		36.8 (34.2-39.4)	2.3 (2.1-2.6)
**West**												
	Maharashtra	0.1(0,0.1)	5.8 (3.6-8.0)	33.3 (0-87.5)	9.3 (7.4-11.2)		10.7 (9.4-12)	19.1 (14.2-24.1)	92.2 (84.1-100.2)		39.5 (35.3-43.7)	4.1 (3.4-4.8)
	Gujarat	0(0,0.1)	1.7 (0-4.0)	0	4.2 (3.5-4.9)		14 (11.5-16.4)	22.1 (16-28.1)	16.4 (15.7-17.2)		49.5 (46.5-52.5)	2.5 (2.1-2.8)
	Goa	0	0	0	2.5 (0.8-4.2)		6.2 (2.5-9.9)	0	191 (169.5-212.4)		28.5 (24.7-32.3)	1.4 (1.2-1.6)
	Dadra and Nagar Haveli	0	0	0	0.4 (0.1-0.9)		0.4 (0.1-0.8)	0	25.3 (34.2-42.9)		114.3 (63.4-165.1)	0.1 (0.1-0.3)
	Daman and Diu	0	0	0	0.4 (0-0.9)		0	0	38.5 (34.2-42.9)		0	0
**South**												
	Andhra Pradesh	0.1	2 (0-4.2)	0	1.2 (1-1.3)		17.3 (14.6-19.9)	7.5 (3-11.9)	108.4 (96.5-120.4)		36.1 (29.9-42.3)	1.5 (1.2-1.7)
	Karnataka	0.1 (0-0.1)	3.2 (0-5.7)	0	1.1 (0.7-1.4)		16.5 (13-20.1)	8.4 (4.2-12.5)	84.6 (76-93.2)		26.9 (24.1-29.7)	2.7 (2.3-3.1)
	Tamil Nadu	0	1.2 (0-2.1)	0	5.4 (4.2-6.7)		11.9 (10.3-13.6)	5.1 (1.8-8.3)	63.3 (60.4-66.3)		43.5 (39-48)	1.9 (1.7-2)
	Kerala	0.3 (0.1-0.4)	4.9 (0-9.9)	0	0.7 (0.5-0.9)		42.4 (26.5-58.4)	2.2 (0.5-4)	71.6 (57.9-85.3)		19.7 (18.5-20.8)	0.6 (0.5-0.7)
	Telangana	0.1 (0-0.1)	3.3 (1.9-4.8)	0	1.2 (1-1.5)		34.4 (22.3-46.5)	10.9 (5.4-16.4)	41.6(38.6,44.7)		38 (33.3-42.7)	2.6 (0.5-4.7)
	Puducherry	0.1 (0-0.2)	0	0	1.1 (0.6-1.7)		2 (0.8-3.2)	0	175.7 (153.3-198)		27.3 (23.1-31.6)	2.7 (2-3.4)
	Andaman and Nicobar Islands	0	0	0	1.1 (0.4-1.7)		0.4 (0.2-0.9)	0	73.4 (58.3-88.5)		70.7 (56.3-85.2)	0
	Lakshadweep	0	0	0	0		0	0	0		0	0
**East**												
	West Bengal	0.1	0.7 (0-1.9)	0(0,0)	1.1 (0.8-1.4)		9.9 (8.6-11.1)	14.5 (8.7-20.2)	23.6 (21.7-25.6)		19.9 (18.8-21.1)	2.4 (2.2-2.6)
	Odisha	0.0	0	0	0.8 (0.6-1.1)		10.5 (8-13)	3.6 (0-7.9)	43.1 (37.8-48.3)		26.3 (23.3-29.3)	0.7 (0.6-0.7)
	Bihar	0.0	0.3 (0-1)	0	0.5 (0.4-0.7)		12.8 (9.7-15.8)	1 (0.3-1.6)	12 (10.6-13.4)		46.8 (40.2-53.3)	0.6 (0.5-0.7)
	Jharkhand	0	0	0	0.4 (0.2-0.5)		13.6 (9.3-17.9)	2.1 (1.5-5.6)	18.6 (16-21.1)		33.9 (28.9-39)	1.6 (1.2-2)
**Northeast**												
	Assam	0.0	0	0	1 (0.6-1.5)		6.4 (4.2-8.7)	4.7 (0.6-10)	41.8 (37.1-46.5)		43.4 (33.4-53.3)	0.1 (0-0.2)
	Tripura	0.0	0	0	2.6 (1.2-3.9)		8.8 (2.5-15)	0	51.7 (44.3-59.1)		46.9 (34.6-59.2)	2.3 (0.6-3.9)
	Manipur	0 (0-0.1)	0	0	0.7 (0.4-1.1)		1.4 (0.1-2.8)	0	37.2 (32.5-41.9)		34.8 (30.4-39.2)	1.5 (0.7-2.4)
	Arunachal Pradesh	0.0	0	0	0.4 (0-0.8)		0.8 (0.1-1.5)	0	64.5 (55.3-73.8)		24.8 (21.4-28.2)	1.1 (0.5-2.7)
	Meghalaya	0.0	0	0	0.1 (0.1-0.2)		1.6 (0.5-2.8)	0	18.7 (15.3-22.1)		26 (22.3-29.6)	2.6 (0.7-4.5)
	Nagaland	0.0	0	0	0.7 (0.2-1.2)		0.5 (0.1-0.9)	0	25.7 (21.5-30)		54.7 (44-65.4)	0.9 (0-1.7)
	Sikkim	0.0	0	0	0.1 (0-0.3)		0 (0-0.1)	0	39.2 (32.8-45.7)		47.2 (34.6-59.7)	2.4 (1.1-3.7)
	Mizoram	0.0	0	0	0.4 (0-0.7)		0.2 (0.1-0.6)	0	13.5 (10.6-16.3)		69.8 (65.7-73.1)	0

^a^CPM: cases per million.

^b^DR: doubling rate.

^c^CFR: case fatality rate.

^d^Values <0.1 are rounded to 0.

### Mobility Indicators and Intramobility Correlation

**Figure 2:**
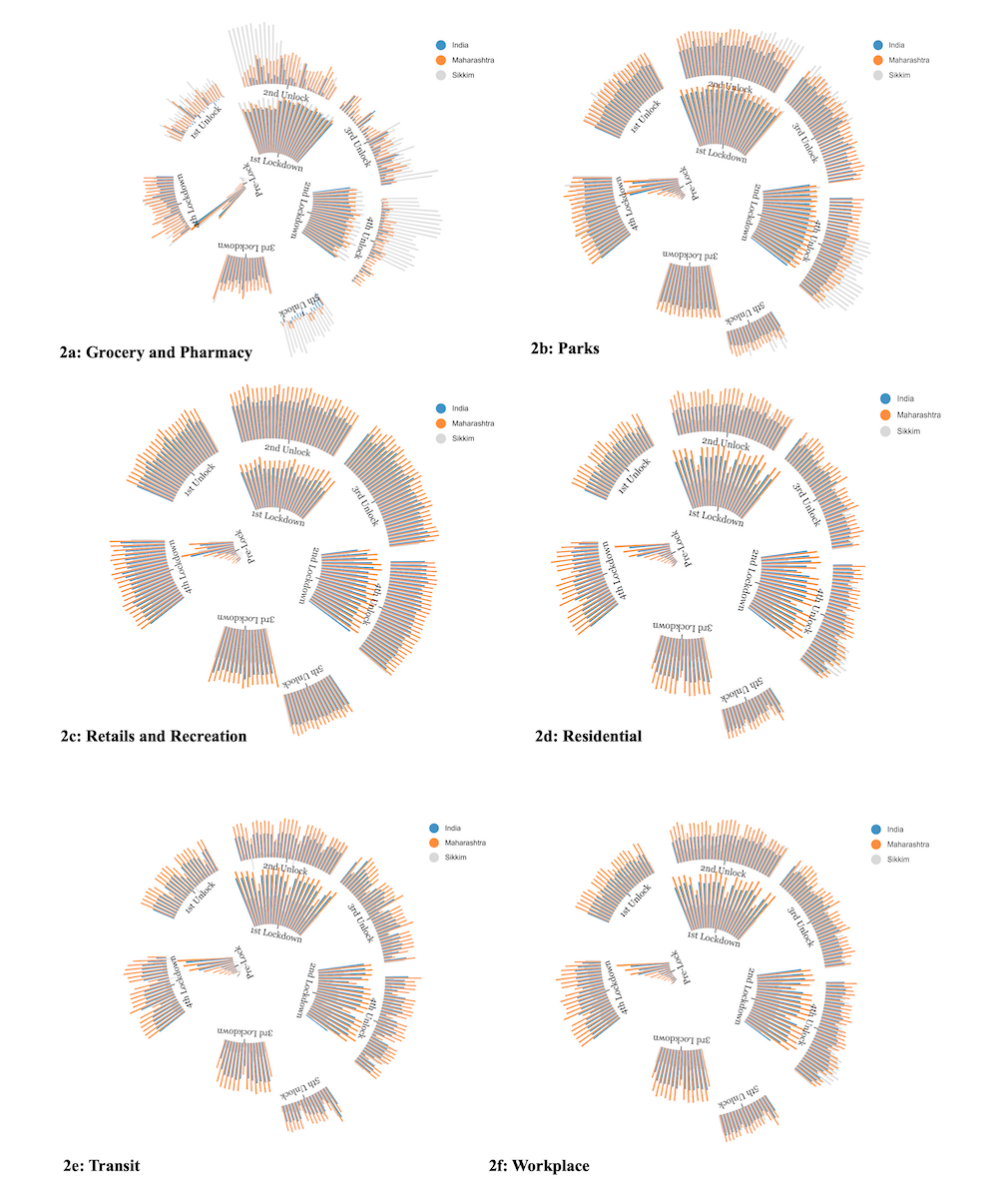
Spiral bar charts displaying the changes in the Google Community Mobility Reports mobility patterns for the states of Sikkim and Maharashtra compared to India across the different phases of lockdown.

Table 3 depicts the changes in the mobility patterns in all 6 categories reported in CMRs for India and for the states of Maharashtra (most cases) and Sikkim (fewest cases). At the national level, mobility in 5 of the 6 categories was reduced during the lockdown period compared to the reference period, with the exception being residential areas. During the lockdown, maximum restrictions were seen at retail and recreation areas, followed by transit, parks, and workplaces. The leading drop of –77.2% (95% CI –78.7% to –75.8%) at the national level occurred for the retail and recreation category during the lockdown. In contrast, residential mobility increased by 24.6% (95% CI 23.4% to 25.8%) during the lockdown. During unlock, the areas with the lowest to highest mobility were residential, groceries and pharmacies, workplaces, transit, parks, and retail and recreation. With the maximum number of cases, Maharashtra State displayed the highest restriction in movement, with a drop of –82.4% (95% CI –83.3% to –81.5%) in the lockdown phase for retail and recreation. Sikkim depicted higher mobility compared to Maharashtra for all 6 categories of places reported in CMRs. The state of Sikkim displayed a drop of –65.4% (95% CI –67% to 63.9%) during lockdown for retail and recreation. The spiral bar charts in Figure 2 display the changes in mobility for the states of Sikkim and Maharashtra as well as at the national level across the different phases of the lockdown. Table 4 exhibits the intramobility correlation. In general, residential mobility was negatively correlated with all other measures of mobility. The magnitude of correlations for the intramobility indicators was comparatively low for the lockdown phase compared to the prelockdown and unlock stages.

**Table 3:** Percentage changes in mobility patterns during different stages of lockdown during the COVID-19 pandemic as per the Google Community Mobility Reports for India (March-October 2020).

Region and mobility indicator		Percentage change in each phase of lockdown, median (95% CI)		
		Prelockdown (before March 25, 2020)	Lockdown (March 25-June 7, 2020)	Unlock (June 8-October 15, 2020)
**India**				
	Retail and recreation	–29.6 (–46.7 to –12.4)	–77.2 (–78.7 to –75.8)	–50.5 (–52.1 to –48.9)
	Transit	–25.7 (–42 to –9.4)	–59.5 (–62.0 to –57.1)	–35.2 (–36.4 to –34)
	Parks	–18.3 (–31.2 to –5.3)	–56.8 (–58.0 to –55.6)	–49.0 (–49.7 to –48.2)
	Workplaces	–21.1 (–36.2 to –5.9)	–51.9 (–55.2 to –48.6)	–28.2 (–29.5 to –27)
	Groceries and pharmacies	–14.0 (–31.4 to 3.4)	–38.0 (–42.4 to –33.6)	–5.7 (–7.2 to –4.1)
	Residential	9.4 (3.8 to 15)	24.6 (23.4 to 25.8)	13.9 (13.6 to 14.3)
**State with highest number of cases at the end of lockdown (Maharashtra)**				
	Retail and recreation	–40.3 (–58.8 to –21.8)	–82.4 (–83.3 to –81.5)	–29.4 (–33.5 to –25.3)
	Transit	–35.6 (–53.8 to –17.3)	–71.3 (–72.8 to –69.8)	–44.3 (–46.2 to –42.4)
	Parks	–30.7 (–45 to –16.5)	–70.4 (–71.5 to –69.2)	–52.2 (–54.1 to –50.2)
	Workplaces	–31.8 (–50.1 to –13.6)	–65.3 (–68 to –62.6)	4.4 (–0.5 to 9.3)
	Groceries and pharmacies	–20.1 (–38.6 to –1.6)	–48 (–50.9 to –45.2)	–52.5 (–55.7 to –49.4)
	Residential	14.5 (7.3 to 21.6)	31.8 (30.6 to 32.9)	22.2 (21.3 to 23.2)
**State with lowest number of cases at the end of lockdown (Sikkim)**				
	Retail and recreation	–8.6 (–23.3 to 6.1)	–65.4 (–67.0 to –63.9)	–52.3 (–55.1 to –49.6)
	Transit	–22.4 (–38.8 to –5.9)	–65.2 (–67.3 to –63)	–54.5 (–57.1 to –51.9)
	Groceries and pharmacies	–12.5 (–30.8 to 5.8)	–48.3 (–52.5 to –44.1)	–39.3 (–43 to –35.6)
	Parks	–16.4 (–29.9 to –2.8)	–43.5 (–44.1 to –42.8)	–43.6 (–44.1 to –43.1)
	Workplaces	0.8 (–8.1 to 9.7)	–20.2 (–23.3 to –17.1)	–15.6 (–17.8 to –13.4)
	Residential	3.6 (0.4 to 6.7)	12.8 (12.1 to 13.5)	13.5 (12.7 to 14.3)

**Table 4:** Intracorrelations between the mobility indicators during the COVID-19 pandemic in India (March-October 2020).

Mobility indicators		Correlation coefficients											
		Prelockdown (before March 25, 2020)				Lockdown (March 25-June 7, 2020)				Unlock (June 8-October 15, 2020)			
		India	Maharashtra	Sikkim	India		Maharashtra	Sikkim	India		Maharashtra	Sikkim	
**Retail and recreation**													
	Groceries and pharmacies	0.60	0.93	0.90	0.40		0.40	0.17	0.37		0.68	0.38	
	Parks	0.96	0.99	0.81	0.45		0.48	0.32	0.69		0.81	0.17	
	Transit	0.97	0.96	0.95	0.38		0.29	0.27	0.77		0.79	0.52	
	Workplaces	0.85	0.82	0.72	0.29		0.20	0.14	0.26		0.43	0.25	
	Residential	–0.91	–0.84	–0.85	–0.44		–0.33	–0.29	–0.30		–0.50	–0.33	
**Groceries and pharmacies**													
	Parks	0.67	0.95	0.85	0.03		–0.07	–0.11	0.46		0.75	0.25	
	Transit	0.56	0.96	0.96	0.91		0.80	0.69	0.47		0.66	0.59	
	Workplaces	0.47	0.82	0.71	0.63		0.51	0.70	–0.08		0.27	0.63	
	Residential	–0.58	–0.81	–0.74	–0.60		–0.40	–0.71	–0.05		–0.34	–0.80	
**Parks**													
	Transit	0.90	0.99	0.87	–0.04		–0.23	–0.15	0.67		0.72	0.10	
	Workplaces	0.77	0.84	0.60	–0.18		–0.27	–0.22	0.10		0.34	0.24	
	Residential	–0.91	–0.85	–0.73	0.07		0.14	0.11	–0.08		–0.36	–0.19	
**Transit**													
	Workplaces	0.88	0.86	0.67	0.70		0.70	0.72	0.35		0.57	0.37	
	Residential	–0.89	–0.84	–0.79	–0.69		–0.61	–0.79	–0.35		–0.64	–0.53	
**Workplaces**													
	Residential	–0.88	–0.99	–0.78	–0.84		–0.85	–0.66	–0.49		–0.74	–0.63	

### Correlation Between Mobility and Epidemiological Indicators

A general trend of a high correlation coefficient between epidemiological and mobility indicators was observed for the lockdown and unlock phases compared to the prelockdown phase. With few exceptions, the correlation coefficients between epidemiological and mobility indicators for India and Maharashtra are similar. The highest correlation for India, Maharashtra, and Sikkim was observed in the unlock stage for retail and recreation and all epidemiological indicators. It was interesting to see a substantial increase in correlation between park visits and epidemiological indicators from the lockdown phase to the unlock phase. Only 7 cases were reported in Sikkim before unlock; therefore, intercorrelations for CFR and recovery are not available during the pre-unlock phases. Table 5 gives details of the correlation coefficients between mobility and epidemiological indicators. Initial exploration indicated that there are substantially high correlations between various epidemiological and Google mobility indicators. Figure 1 displays the cumulative rise in the frequency of cases with the mobility indicators. There was a rapid surge in the number of cases in the unlock phase after flat linear growth up to the lockdown stage.

**Table 5:** Intercorrelation between the mobility and epidemiological indicators during the COVID-19 pandemic in India (March-October 2020).

Mobility and epidemiological indicators			Correlation coefficients																					
			Prelockdown (before March 25, 2020)								Lockdown (March 25-June 7, 2020)							Unlock (June 8-October 15, 2020)						
			India		Maharashtra		Sikkim			India			Maharashtra		Sikkim		India			Maharashtra	Sikkim			
**Retail and recreation**																								
	Doubling rate	0.00		0.11		—^a^		0.29				0.29		0.15		0.72			0.66		0.27			
	Case fatality rate	–0.49		-0.55		—		–0.21				–0.33		—		–0.64			–0.55		0.37			
	Recovery	0.02		—		—		0.32				0.30		—		0.63			0.64		0.12			
**Groceries and pharmacies**																								
	Doubling rate	0.00		0.16		—		0.78				0.62		0.19		0.15			0.43		–0.01			
	Case fatality rate	–0.44		–0.37		—		–0.69				–0.41		—		–0.05			–0.35		–0.16			
	Recovery	–0.14		—		—		0.85				0.76		—		0.00			0.37		–0.30			
**Parks**																								
	Doubling rate	0.05		0.11		—		–0.06				–0.13		0.05		0.51			0.59		0.02			
	Case fatality rate	–0.41		–0.57		—		0.14				–0.06		—		–0.44			–0.48		–0.07			
	Recovery	–0.06		—		—		–0.07				–0.20		—		0.43			0.55		–0.05			
**Transit**																								
	Doubling rate	–0.02		0.11		—		0.79				0.62		0.14		0.59			0.60		0.13			
	Case fatality rate	–0.53		–0.55		—		–0.68				–0.39		—		–0.51			–0.49		0.09			
	Recovery	0.04		—		—		0.87				0.80		—		0.47			0.55		–0.08			
**Workplaces**																								
	Doubling rate	–0.09		0.07		—		0.59				0.46		0.13		0.35			0.35		–0.10			
	Case fatality rate	–0.40		–0.55		—		–0.56				–0.23		—		–0.37			–0.35		–0.06			
	Recovery	–0.02		—		—		0.70				0.57		—		0.35			0.38		–0.31			
**Residential**																								
	Doubling rate	0.05		–0.07		—		–0.55				–0.40		–0.16		–0.35			–0.41		0.02			
	Case fatality rate	0.36		0.57		—		0.51				0.23		—		0.30			0.34		0.08			
	Recovery	0.06		—		—		–0.65				–0.48		—		–0.25			–0.36		0.31			

^a^Not applicable.

## Discussion

We used the CMRs provided by Google to assess the national and subnational patterns of mobility before, during, and after the COVID-19 pandemic lockdown enforced by the government of India and their correlations with disease severity. There are specific critical findings in our study. First, there were marked interstate variations in the disease burden during the three phases of our study period. By the end of the lockdown phase, although the CPM and DR continued to increase, disease severity, as depicted by the CFR, started to decrease. The CMR data depicted that mobility decreased during the lockdown and then increased again during the unlock phase. We observed intramobility solid patterns among the 6 mobility indicators. Residential mobility was seen to be inversely associated with mobility in public places. A significant correlation was seen between mobility and epidemiological indicators.

Inter- and intramobility networks play significant roles in disease transmission dynamics in the modern era [44]. We observed wide subnational variations in the disease burden, as depicted by various epidemiological indicators used in the study. The state of Maharashtra was among the most greatly affected states in the country. This can be attributed to its large population size, as it is the second most populous state in India after Uttar Pradesh. Moreover, COVID-19 was a relatively urban phenomenon during the study period, and Maharashtra is one of India's most urbanized states (more than 50% urbanized). More than half of the COVID-19 cases in Maharashtra were reported from four major cities: Mumbai, Thane, Pune, and Nagpur. In contrast, the proportion of urbanization in other populous states such as Uttar Pradesh is 22%. Also, Maharashtra attracts more people from other states for education and jobs; hence, it has a very high population density. This demographic profile has a significant impact on the COVID-19 transmission dynamics. However, if we consider total CPM, many other states, such as Goa, Delhi, and Andhra Pradesh, reported more cases than Maharashtra by the end of lockdown. Also, any state with an efficient and sensitive surveillance system in place will detect more cases during an epidemic. Maharashtra has always been among the top performers in India in this regard [45]. On the other hand, the state of Sikkim depicted the minimum number of cases throughout the country. Sikkim is a remote state in a hilly area, with a small population size and lower population density, fewer migrations, more rural areas, and less interstate trade and transit; this explains its lower number of cases during the study period.

In our study, residential mobility increased during the lockdown. It also correlated negatively with other measures of mobility. These findings are consistent with studies conducted by Saha et al [34,46], which found that people stayed at home during the lockdown. The mobility trends display that mobility started to decrease even before the government implemented the lockdown measure. Although legal enforcement was the prime reason for reducing mobility, people also restricted their movements voluntarily and avoided crowded places due to apprehensions regarding the disease [47-49]. Mobility in other places was reduced during the lockdown and then gradually rescaled during the unlock phase. This pattern is coherent with those in other studies, which found that people rescheduled or canceled travel and transport plans in the wake of public health emergencies [50,51].

We used the Kendall tau correlation to quantify the relationship between mobility and epidemiological indicators, as this correlation is more robust and consistent for nonnormal data. The mobility indicators depicted strong correlations with epidemiological characteristics for both the lockdown and unlock phases. This trend is consistent with many theoretical studies that have predicted the role of mobility for infectious diseases [10,52,53]. Previous studies have demonstrated the utility of mobility in the spread of COVID-19 data globally [31,54]. Our analysis indicates that mobility is a potential metric to monitor and predict disease outbreaks. However, the statistical analysis is exploratory and univariate and requires rigorous statistical evaluation before mobility can be adopted as an indicator of surveillance.

Moreover, our models depicted wide interstate variations in mobility patterns. We discussed variations only in the states of Maharashtra and Sikkim, as they reported the maximum and minimum numbers of cases, respectively, at the end of lockdown to understand the relationship between mobility and disease dynamics. Addressing diseases such as COVID-19 from a mathematical perspective can reveal the internal pattern and potential structure of pandemic control, and it can help provide insights into the transmission dynamics of such diseases and the potential role of different public health intervention strategies [55]. Lockdown interventions to prevent the spread of infection lead to different patterns of mobility. However, lockdown measures only serve their purpose when they ate strictly enforced. Our data suggest that the growth trajectory for the rise in cases was linear compared to the steep trajectory post lockdown. Many authors have previously discussed the impact of lockdown in controlling the spread of COVID-19 in India [56,57]. However, lockdown measures to save lives were recommended and championed by the WHO and other leading agencies. There are numerous discussions and debates in the literature regarding the appropriateness of total lockdown measures [58-60]. A group of medical researchers published the “Great Barrington Declaration,” in which they emphasized the concept of “Focused Protection” as an alternative to lockdowns [61]. Simultaneously, other researchers disagreed and called for strict measures until a vaccine became available; they published the “John Snow Memorandum” [62]. However, it may take a long time to assess the overall strengths and shortcomings of the lockdown.

This study is the first pan-Indian empirical study quantifying the role of mobility in disease transmission. However, there are some obvious limitations to our study. The major limitation is the dynamic nature of COVID-19 and the mobility patterns. Therefore, it is challenging to obtain robust estimates unless disease transmission stabilizes. Moreover, an ecological study uses normative mobility data; this study may thus be impacted by ecological fallacy. The disease infection rate varies per gender, accessibility to health care, and literacy level; however, the data for the current study limit its generalization to these subgroups.

Similarly, no attempt can be made to examine the psychological and sociological issues affecting mobility. The data used by Google to generate the mobility estimates may have questionable concordance with the actual mobility rates. Mobile phones may not reflect the actual mobility in the community, especially in rural areas, where GPS-enabled smartphones are not used by many people. Similarly, apprehensions about data misuse may prevent many smartphone users from using maps, undermining the actual estimates. Less frequent GPS usage may be a reason why we could not find intermobility patterns for Sikkim in our analysis. Finally, per CMR, baseline dates do not account for the seasonality of movements. The lack of accounting of seasonality may also affect the accuracy and precision of estimates. Moreover, Google’s CMR data do not directly equate to some specific COVID-19 control measures. We could not assess the reasons underlying the patterns observed in mobility.

To conclude, we can use mobile-based open-source mobility data to assess the effectiveness of social distancing. CMR data depicted an association between community mobility with disease severity indicators. We suggest that data related to community mobility can be of utility in future COVID-19 modeling studies. With the declaration of COVID-19 as a pandemic, mobility levels declined, which can be primarily attributed to legal enforcement or increased fear of disease leading to personal behavioral changes. Google’s CMR depicts the effect of these measures on community movement. CMR can provide an effective tool for the authorities to evaluate the timing and impact of social distancing efforts, mainly related to movement restrictions. We recommend using these data whenever applicable to supplement the existing surveillance methods in any country. This approach does not involve any additional cost and can provide quick action points about the adherence to social distancing measures. This method can be used to forecast mass movements during nonpandemic conditions, such as the famous gatherings during Kumbh Mela in India, and can help us assess preparedness accordingly. An attempt can also be made to forecast mass movements, which is needed to make informed decisions. With the increase in mobile internet usage, the real-time data method is expected to increase accuracy. Future studies should focus on establishing the cultural, social, and economic issues that are responsible for some of the differences in adherence to social distancing measures.

## Appendix

Multimedia Appendix 1 Summary of mobility reports generated by Google to combat the COVID-19 pandemic and snapshot of the process to download community mobility reports and data.

## Abbreviations

CFR: case fatality rate
CPM: cases per million
DR: doubling rate
MERS: Middle East respiratory system
NPI: nonpharmaceutical intervention
SARS: severe acute respiratory syndrome
WHO: World Health Organization

## References

[1] Christiansen J (2018). Global infections by the numbers. Scentific Am.

[2] Bloom DE, Cadarette D (2019). Infectious disease threats in the twenty-first century: strengthening the global response. Front Immunol.

[3] Smith KF, Goldberg M, Rosenthal S, Carlson L, Chen J, Chen C, Ramachandran S (2014). Global rise in human infectious disease outbreaks. J R Soc Interface.

[4] Coronavirus. World Health Organization.

[5] Anastassopoulou C, Russo L, Tsakris A, Siettos C (2020). Data-based analysis, modelling and forecasting of the COVID-19 outbreak. PLoS One.

[6] Coronavirus disease (COVID-19) situation reports. World Health Organization.

[7] COVID19-India API.

[8] Askitas N, Tatsiramos K, Verheyden B (2021). Estimating worldwide effects of non-pharmaceutical interventions on COVID-19 incidence and population mobility patterns using a multiple-event study. Sci Rep.

[9] Ray D, Subramanian S (2020). India's lockdown: an interim report. Indian Econ Rev.

[10] Bengtsson L, Gaudart J, Lu X, Moore S, Wetter E, Sallah K, Rebaudet S, Piarroux R (2015). Using mobile phone data to predict the spatial spread of cholera. Sci Rep.

[11] Kraemer MUG, Yang C, Gutierrez B, Wu C, Klein B, Pigott DM, du Plessis Louis, Faria Nuno R, Li Ruoran, Hanage William P, Brownstein John S, Layan Maylis, Vespignani Alessandro, Tian Huaiyu, Dye Christopher, Pybus Oliver G, Scarpino Samuel V, Open COVID-19 Data Working Group (2020). The effect of human mobility and control measures on the COVID-19 epidemic in China. Science.

[12] Barmak DH, Dorso CO, Otero M, Solari HG (2011). Dengue epidemics and human mobility. Phys Rev E Stat Nonlin Soft Matter Phys.

[13] Charu V, Zeger S, Gog J, Bjørnstad Ottar N, Kissler S, Simonsen L, Grenfell BT, Viboud C (2017). Human mobility and the spatial transmission of influenza in the United States. PLoS Comput Biol.

[14] Peak CM, Wesolowski A, Zu Erbach-Schoenberg E, Tatem AJ, Wetter E, Lu X, Power D, Weidman-Grunewald E, Ramos S, Moritz S, Buckee CO, Bengtsson L (2018). Population mobility reductions associated with travel restrictions during the Ebola epidemic in Sierra Leone: use of mobile phone data. Int J Epidemiol.

[15] Wesolowski A, Eagle N, Tatem AJ, Smith DL, Noor AM, Snow RW, Buckee CO (2012). Quantifying the impact of human mobility on malaria. Science.

[16] Yang W, Wen L, Li S, Chen K, Zhang W, Shaman J (2017). Geospatial characteristics of measles transmission in China during 2005−2014. PLoS Comput Biol.

[17] Grenfell BT, Bjørnstad O N, Kappey J (2001). Travelling waves and spatial hierarchies in measles epidemics. Nature.

[18] Moghadas SM, Shoukat A, Fitzpatrick MC, Wells CR, Sah P, Pandey A, Sachs JD, Wang Z, Meyers LA, Singer BH, Galvani AP (2020). Projecting hospital utilization during the COVID-19 outbreaks in the United States. Proc Natl Acad Sci U S A.

[19] Stang A, Stang M, Jöckel Karl-Heinz (2020). Estimated use of intensive care beds due to COVID-19 in Germany over time. Dtsch Arztebl Int.

[20] Gensini GF, Yacoub MH, Conti AA (2004). The concept of quarantine in history: from plague to SARS. J Infect.

[21] Tognotti E (2013). Lessons from the history of quarantine, from plague to influenza A. Emerg Infect Dis.

[22] Jang WM, Jang DH, Lee JY (2020). Social distancing and transmission-reducing practices during the 2019 coronavirus disease and 2015 Middle East respiratory syndrome coronavirus outbreaks in Korea. J Korean Med Sci.

[23] Ferguson NM, Cummings DAT, Fraser C, Cajka JC, Cooley PC, Burke DS (2006). Strategies for mitigating an influenza pandemic. Nature.

[24] Christaki E (2015). New technologies in predicting, preventing and controlling emerging infectious diseases. Virulence.

[25] Tom-Aba D, Nguku PM, Arinze CC, Krause G (2018). Assessing the concepts and designs of 58 mobile apps for the management of the 2014-2015 West Africa Ebola outbreak: systematic review. JMIR Public Health Surveill.

[26] Nsoesie EO, Kluberg SA, Mekaru SR, Majumder MS, Khan K, Hay SI, Brownstein JS (2015). New digital technologies for the surveillance of infectious diseases at mass gathering events. Clin Microbiol Infect.

[27] Eysenbach G (2003). SARS and population health technology. J Med Internet Res.

[28] Salathé Marcel (2018). Digital epidemiology: what is it, and where is it going?. Life Sci Soc Policy.

[29] Fond G, Gaman A, Brunel L, Haffen E, Llorca P (2015). Google Trends: Ready for real-time suicide prevention or just a Zeta-Jones effect? An exploratory study. Psychiatry Res.

[30] Verma M, Kishore K, Kumar M, Sondh AR, Aggarwal G, Kathirvel S (2018). Google search trends predicting disease outbreaks: an analysis from India. Healthc Inform Res.

[31] Sulyok M, Walker M (2020). Community movement and COVID-19: a global study using Google's Community Mobility Reports. Epidemiol Infect.

[32] Wang HY, Yamamoto N (2020). Using a partial differential equation with Google Mobility data to predict COVID-19 in Arizona. Math Biosci Eng.

[33] Cot C, Cacciapaglia G, Sannino F (2021). Mining Google and Apple mobility data: temporal anatomy for COVID-19 social distancing. Sci Rep.

[34] Saha J, Barman B, Chouhan P (2020). Lockdown for COVID-19 and its impact on community mobility in India: an analysis of the COVID-19 Community Mobility Reports, 2020. Child Youth Serv Rev.

[35] Middelburg RA, Rosendaal FR (2020). COVID-19: How to make between-country comparisons. Int J Infect Dis.

[36] GRID COVID-19 Study Group (2020). Combating the COVID-19 pandemic in a resource-constrained setting: insights from initial response in India. BMJ Glob Health.

[37] COVID-19 lockdown in India. Wikipedia.

[38] COVID-19 Community Mobility Reports. Google.

[39] Overview - Community Mobility Reports Help. Google.

[40] Sornette D, Mearns E, Schatz M, Wu K, Darcet D (2020). Interpreting, analysing and modelling COVID-19 mortality data. Nonlinear Dyn.

[41] Patel SB, Patel P (2020). Doubling time and its interpretation for COVID 19 cases. Natl J Commmunity Med.

[42] (2020). Estimating mortality from COVID-19. World Health Organization.

[43] (2020). State/UT wise Aadhaar saturation (overall) - all age groups. Goverment of India.

[44] Koopman J (2004). Modeling infection transmission. Annu Rev Public Health.

[45] Health performance. Government of India.

[46] Saha J, Chouhan P (2021). Lockdown and unlock for the COVID-19 pandemic and associated residential mobility in India. Int J Infect Dis.

[47] Sadique MZ, Edmunds WJ, Smith RD, Meerding WJ, de Zwart O, Brug J, Beutels P (2007). Precautionary behavior in response to perceived threat of pandemic influenza. Emerg Infect Dis.

[48] Barr M, Raphael B, Taylor M, Stevens G, Jorm L, Giffin M, Lujic S (2008). Pandemic influenza in Australia: using telephone surveys to measure perceptions of threat and willingness to comply. BMC Infect Dis.

[49] Liao Q, Cowling BJ, Wu P, Leung GM, Fielding R, Lam WWT (2015). Population behavior patterns in response to the risk of influenza A(H7N9) in Hong Kong, December 2013-February 2014. Int J Behav Med.

[50] Goodwin R, Gaines SO, Myers L, Neto F (2010). Initial psychological responses to swine flu. Int J Behav Med.

[51] Anke J, Francke A, Schaefer L, Petzoldt T (2021). Impact of SARS-CoV-2 on the mobility behaviour in Germany. Eur Transp Res Rev.

[52] Wesolowski A, Buckee CO, Engø-Monsen Kenth, Metcalf CJE (2016). Connecting mobility to infectious diseases: the promise and limits of mobile phone data. J Infect Dis.

[53] Eshraghian EA, Ferdos SN, Mehta SR (2020). The impact of human mobility on regional and global efforts to control HIV transmission. Viruses.

[54] Yilmazkuday H Stay-at-home works to fight against COVID-19: international evidence from Google mobility data. SSRN Journal..

[55] Xiang Y, Jia Y, Chen L, Guo L, Shu B, Long E (2021). COVID-19 epidemic prediction and the impact of public health interventions: a review of COVID-19 epidemic models. Infect Dis Model.

[56] Ambikapathy B, Krishnamurthy K (2020). Mathematical modelling to assess the impact of lockdown on COVID-19 transmission in India: model development and validation. JMIR Public Health Surveill.

[57] Meo S, Abukhalaf A, Alomar A, AlMutairi F, Usmani A, Klonoff D (2020). Impact of lockdown on COVID-19 prevalence and mortality during 2020 pandemic: observational analysis of 27 countries. Eur J Med Res.

[58] Jribi S, Ben Ismail H, Doggui D, Debbabi H (2020). COVID-19 virus outbreak lockdown: what impacts on household food wastage?. Environ Dev Sustain.

[59] Lenzer J (2020). Covid-19: Experts debate merits of lockdowns versus "focused protection". BMJ.

[60] Siqueira CDS, Freitas YD, Cancela MDC, Carvalho M, Oliveras-Fabregas A, de Souza Dyego Leandro Bezerra (2020). The effect of lockdown on the outcomes of COVID-19 in Spain: an ecological study. PLoS One.

[61] Martin K, Gupta S, Bhattacharya J (2020). Great Barrington Declaration.

[62] Alwan NA, Burgess RA, Ashworth S, Beale R, Bhadelia N, Bogaert D, Dowd J, Eckerle I, Goldman LR, Greenhalgh T, Gurdasani D, Hamdy A, Hanage WP, Hodcroft EB, Hyde Z, Kellam P, Kelly-Irving M, Krammer F, Lipsitch M, McNally A, McKee M, Nouri A, Pimenta D, Priesemann V, Rutter H, Silver J, Sridhar D, Swanton C, Walensky RP, Yamey G, Ziauddeen H (2020). Scientific consensus on the COVID-19 pandemic: we need to act now. Lancet.

