# Supplementary material for: Exploring the Utility of Google Mobility Data During the COVID-19 Pandemic in India: Digital Epidemiological Analysis
Source: JMIR Public Health Surveill. 2021 Aug 30;7(8):e29957. doi: 10.2196/29957 (PMC8407437; doi:10.2196/29957)
Supplement: Multimedia Appendix 1 [file publichealth_v7i8e29957_app1.docx]

**Appendix**

**Appendix 1: Summary of mobility reports generated by Google to combat the COVID-19 pandemic.**

| 1. COVID-19 Community Mobility Reports can be accessed for 135 countries at [Google Mobility Report](https://d.docs.live.net/Users/kamalkishore/Downloads/(https:/www.google.com/covid19/mobility/)) as of date 15.10.2020. |
| --- |
| 1. Mobility data is almost daily updated for six important mobility events named grocery and pharmacy, parks, transit stations, retail and recreation, residential and workplaces. |
| 1. The data is normalized, anonymized, and aggregated at city, country, or region level view for change in mobility patterns. |
| 1. The baseline value to capture and compare mobility over time is the median mobility value for the day between 3 January - 6 February, 2020. |
| 1. There are gaps in the data as data did not meet the quality and privacy threshold to meet anonymity standards. |

**Appendix 2: Snapshot of the process to download community mobility reports and data**

| 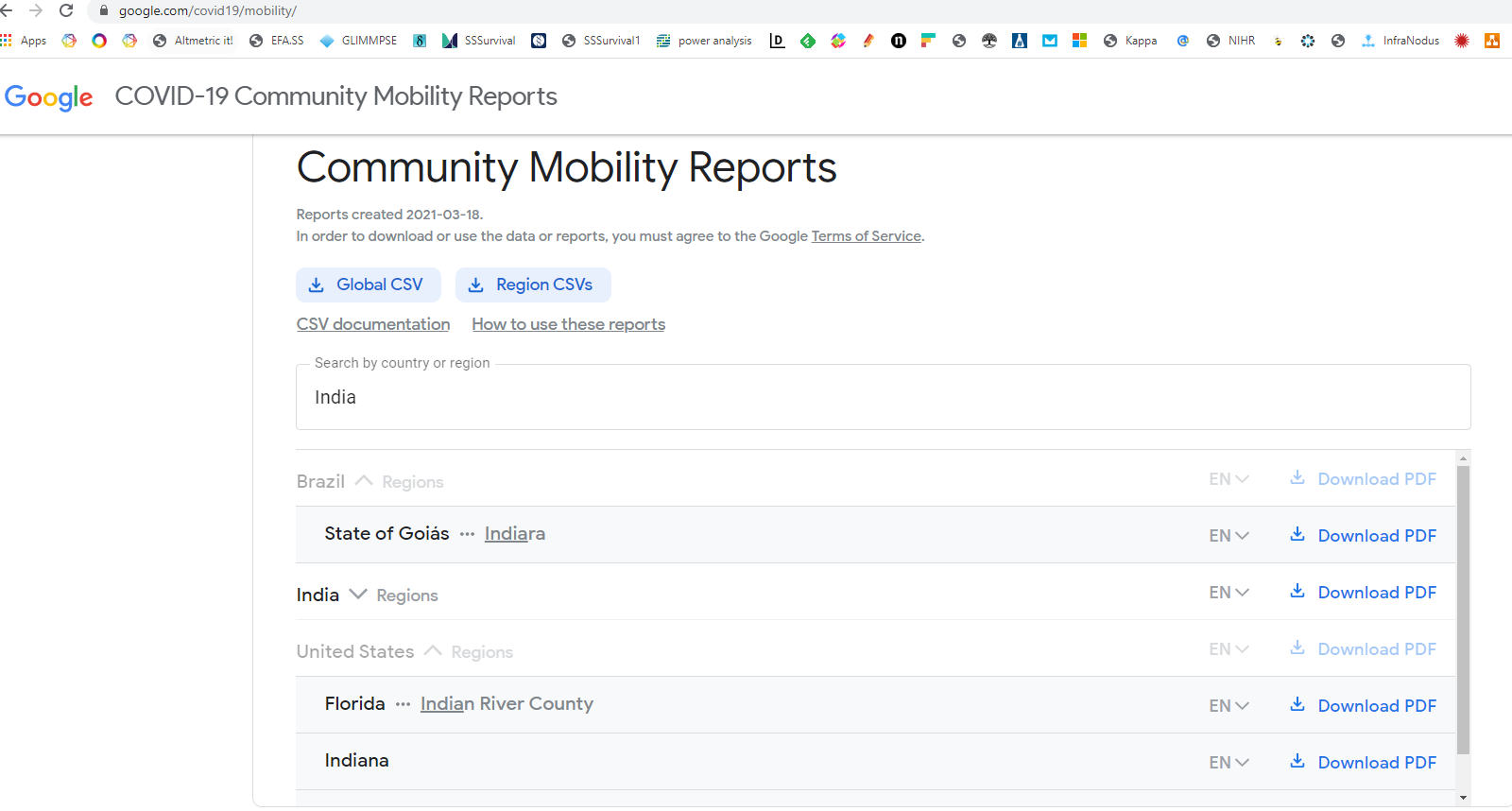  Links to data and documentation  Download Data  Date of report creation  URL: Unit Remote Location Address  Enter name of Country or region of interest |
| --- |
| 2 (b): Generate region-specific report by clicking on the inverted caret sign |
| 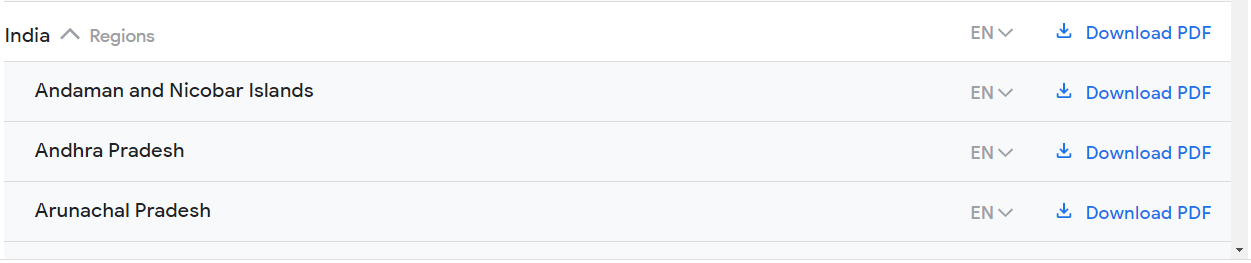 |
